# Assessment of interobserver reliability and predictive values of CT semiquantitative and severity scores in COVID lung disease

**DOI:** 10.1186/s43055-021-00523-z

**Published:** 2021-06-18

**Authors:** Dhilip Andrew, Karthik Shyam, Soumya Cicilet, Jovis Johny

**Affiliations:** Department of Radiology, St. John’s Medical College Hospital, Sarjapur Road, Bangalore, Karnataka 560034 India

**Keywords:** COVID, Pneumonia, HRCT, Score, Reliability

## Abstract

**Background:**

The coronavirus disease (COVID-19), caused by severe acute respiratory syndrome coronavirus 2 (SARS-CoV-2), and first reported in December 2019 at Wuhan, China, has since then progressed into an ongoing global pandemic. The primary organ targeted by the virus is the pulmonary system, leading to interstitial pneumonia and subsequent oxygen dependency and morbidity. Computed tomography (CT) has been used by various centers as an imaging modality for the assessment of severity of lung involvement in individuals. Two popular systems of scoring lung involvement on CT are CT semiquantitative score (SQ) and CT severity score (CT-SS), both of which assess extent of pulmonary involvement by interstitial pneumonia and are partly based upon subjective evaluation. Our cross-sectional observational study aims to assess the interobserver reliability of these scores, as well as to assess the statistical correlation between the respective CT scores to severity of clinical outcome.

**Results:**

Both the SQ and CT-SS scores showed an excellent interobserver reliability (ICC 0.91 and 0.93, respectively, *p* < 0.05). The CT-SS was marginally more sensitive (99.2%) in detecting severe COVID pneumonia than SQ (86.5%). The positive predictive value of SQ (98.3%) is more than CT-SS (78%) for detecting severe disease. The similarity of interobserver reliability obtained for both scores reiterates the respective cutoff CT scores proposed by the above systems, as 18 for SQ and 19.5 for CT-SS.

**Conclusion:**

Both the SQ and CT-SS scores display excellent interobserver reliability. The CT-SS was more sensitive in detecting severe COVID pneumonia and may thus be preferred over the SQ as an initial radiological tool in predicting severity of infection.

## Background

The coronavirus disease (COVID-19) is caused by severe acute respiratory syndrome coronavirus 2 (SARS-CoV-2). The outbreak was first reported in December 2019 at Wuhan, China, since then the disease has progressed into an ongoing global pandemic [1]. As of December 15th 2020, there have been 71,581,532 confirmed cases and 1,618,374 deaths [2]. The disease affects patients of all ages; however, they can progress rapidly in elderly patients and patients with comorbidities (such as hypertension, chronic heart disease, chronic obstructive pulmonary disease, and diabetes). Elderly patients and those with comorbidities can present with multiple systemic complications secondary to the disease [3, 4]. The current gold standard for diagnosis of COVID-19 is real-time polymerase chain reaction (RT-PCR) [5]. Role of radiological imaging in screening and diagnosis of COVID is much debated, with recommendations against and supportive of chest computed tomogram (CT) as first line of screening. However, according to the guidelines of European Society of Radiology and European Society of Thoracic Imaging the limited role of chest CT in resource limited set up is recognized [6, 7]. Chest imaging plays an important role in working up and staging of COVID-19: it can help stratify cases of COVID-19 based on imaging findings and find patients most at risk of adverse clinical outcomes [7–9]. Chest CT has a higher sensitivity(86–98%) and lower false-negative rate compared to RT-PCR [10–12].

The more commonly reported CT chest findings in COVID-19 patients are ground glass opacities (GGO), reticular opacities, and consolidation. Less common CT findings were subpleural lines, crazy paving sign (ground glass opacities superimposed with interlobular lines and septal thickening), and bronchial wall thickening. Atypical CT findings are pleural effusion and mediastinal lymphadenopathy [6, 9, 13–16]. The predominant distribution of findings are bilateral, peripheral, subpleural, posterior, and basal in distribution [9, 14]. The CT findings observed in COVID-19 are not specific and overlap with a number of other conditions [12, 17, 18]. Given the need for a common consensus and language for reporting the Chest CT findings in COVID, CT reporting guidelines by RSNA (Radiology Society of North America) and CO-RADS by Dutch Radiological Society were proposed. These guidelines provided the radiologist with terminology and language required for reporting the findings and thus helped improving interobserver agreement. The interobserver agreement for RSNA score and CO-RADS were excellent (Fliess kappa value of 0.871 and 0.876) [18].

According to study by Jiong et al., chest CT could be used to evaluate the clinical severity of the disease since there were correlation between lung involvement, laboratory markers, and clinical symptoms [9]. Various CT scoring systems have been proposed to assess the lung involvement, the two most commonly used scores being the semiquantitative (SQ) score and CT severity score (CT-SS) [19, 20]. CT scoring systems have been used to predict the clinical outcome like mortality and chart the prognosis of the patient [21, 22]. Given the vital role of CT scoring system in monitoring the clinical prognosis of the COVID-19 patient, there is need for a CT score with good interobserver agreement and correlation with clinical severity.

### Aims and objectives

To compare CT-SS and SQ scores and find the score with the least interobserver variation for determining the lung involvement in COVID-19 pneumonia, and to determine the correlation between lung involvement and clinical severity of COVID pneumonia.

## Method

A cross-sectional study was undertaken at a tertiary care referral academic center designated for management of COVID-19 patients. The study was conducted between June 2020 and December 2020. Institutional ethical clearance was obtained and informed consent was waived.

### Patient selection

A total of 120 adults (≥ 18 years old) hospitalized with laboratory confirmed COVID-19 infection were included in the study. Inclusion criteria included patients who have newly tested positive for COVID-19 (RT-PCR) at the time of admission or after admission, and had a CT scan of the chest performed during admission. Exclusion criteria included patients preliminarily treated for COVID-19 in another hospital and individuals with re-infection with COVID-19.

### Clinical data collection

Clinical and laboratory data such as demographic details, symptoms at presentation, examination findings, arterial blood gas findings, and CT chest findings were collected. According to the following criteria, patients were clinically categorized into mild and severe pneumonia according to guidelines laid by Chinese Center for Disease Control [23]. Patients were categorized with *mild disease* (mild symptoms without dyspnea, respiratory, frequency < 30/min; blood oxygen saturation (SpO2) > 93%; PaO2/FiO2 ratio ≥ 300 mmHg), *severe disease* (dyspnea, respiratory frequency ≥ 30/min, SpO2 ≤ 93%, PaO2/FiO2 ratio < 300 mmHg, and/or lung infiltrates on X-ray > 50% within 24 to 48 h), and *critical disease* (patients with adult respiratory distress syndrome or respiratory failure/acute respiratory distress syndrome (ARDS), septic shock, and multiple organ dysfunction (MOD) or multi-organ failure (MOF). The patients in the critical disease group were categorized under the severe disease group for the purpose of our study.

### Image acquisition

Imaging was carried out using a helical 64 multi-slice CT scanner. Images were acquired in a single breath hold spanning entire chest from the diaphragmatic domes to lung apices. CT scan parameters were as follows: X-ray tube parameters 120 kVp, 250 mAs; rotation time 0.5 s; pitch 1.0; section thickness 5 mm; intersection space 5 mm. Images were reconstructed to 0.625 mm thickness sections using the soft tissue and lung kernels. Images were analyzed using standard viewing software.

### Image analysis

**Fig. 1:**
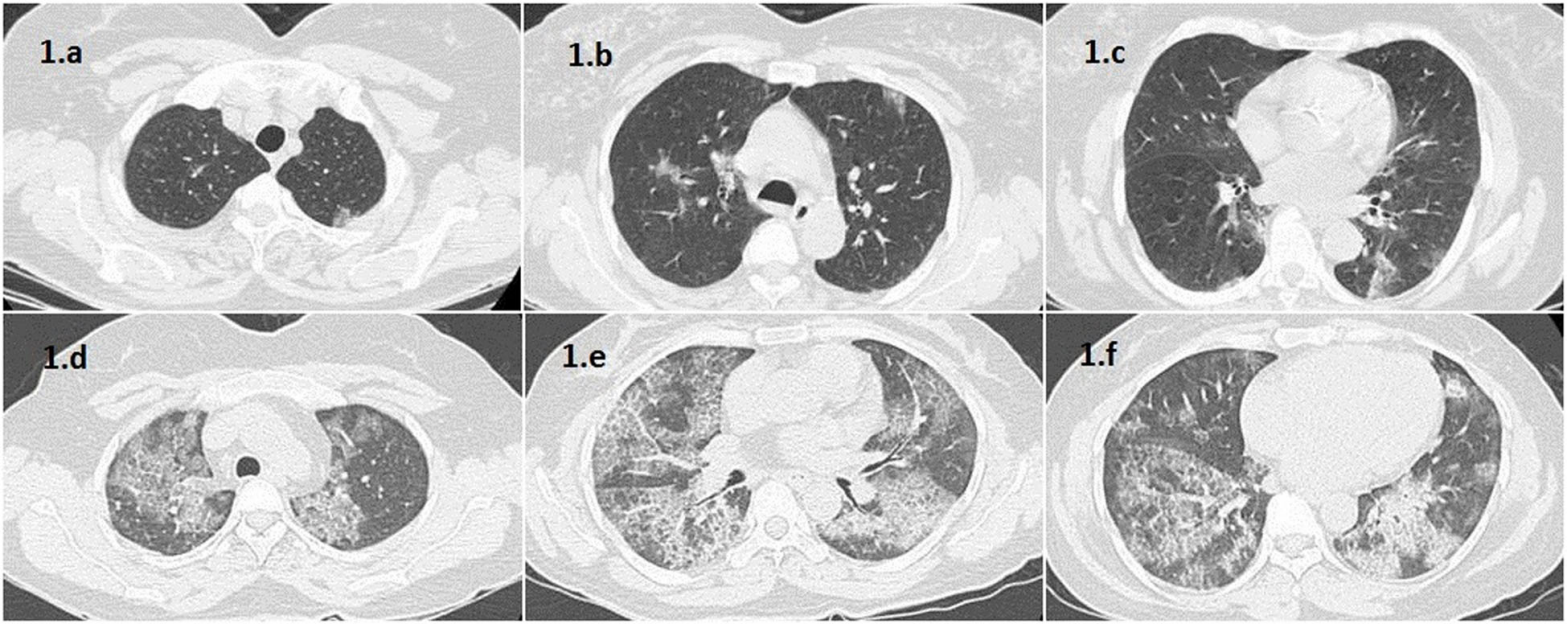
**a**-**c** 45-year-old female with clinically mild COVID and SQS of 8, **d**-**f** 52-year-old male with clinically severe COVID and SQS of 22

**Fig 2:**
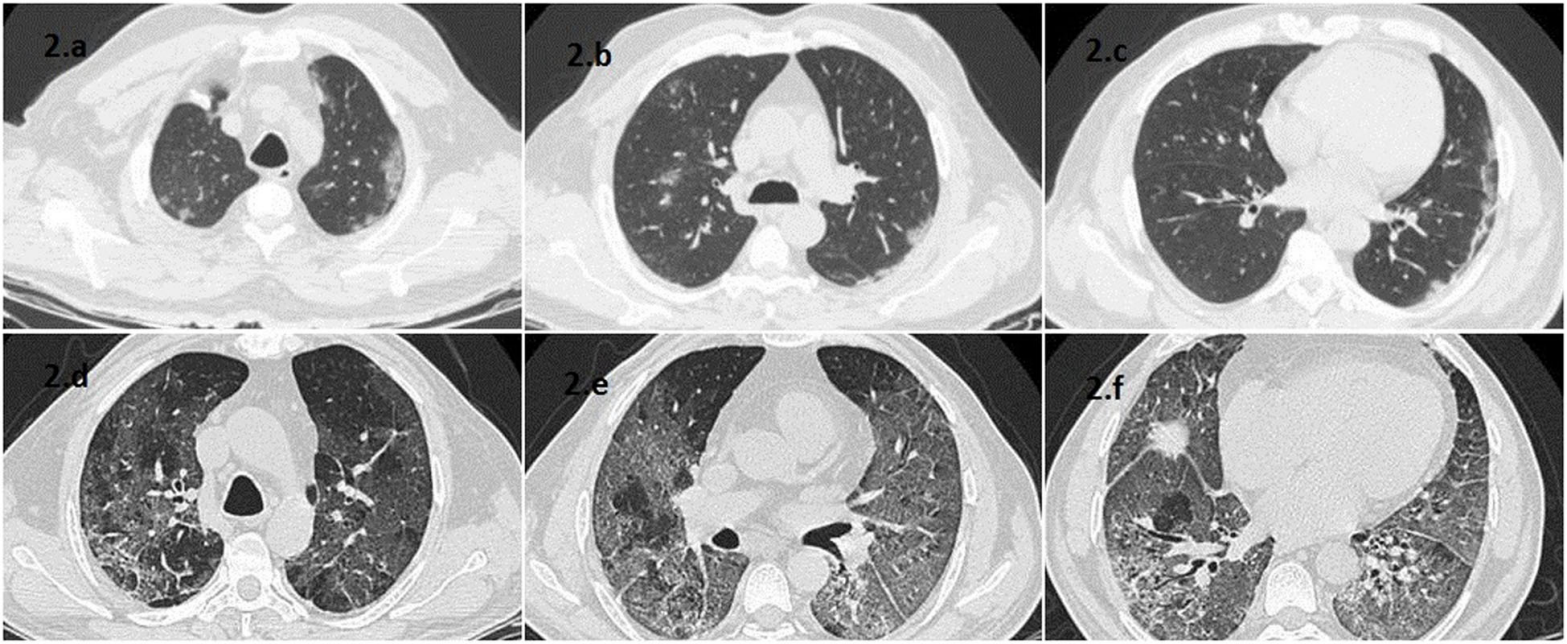
**a**-**c** 50-year-old male with clinically mild COVID pneumonia and CTSS of 11, **d**-**f** 45-year-old male with clinically severe COVID pneumonia and CTSS of 32

Images were separately evaluated by two radiologists with 4 and 10 years of experience in thoracic CT interpretation, while being blinded to the clinical details. Findings such as ground glass opacities, consolidation, and crazy paving were recorded using the standard nomenclature defined by the Fleischner Society glossary [24]. CT-SS and SQ scores for each patient were calculated. In case of CT-SS, lung opacities in all of the 20 segments of lung were evaluated and a score of either 0, 1, or 2 were given if 0%, less than 50%, or 50% or more of each segment was involved, respectively. Lung segments were determined according to the system utilized by Yang et al. [20], where each lobe is divided into its constituent segments, and the lingular segment being divided into superior and inferior subsegments. The final CT-SS score is the sum of these individual scores with a possible range from 0 to 40 [20] (Fig. 1). A score of 19 or more was categorized as radiologically severe. In case of SQ score, lung opacities in all the 5 lobes of the lung were evaluated and scored individually: 0 if no involvement, 1 if < 5% involved, 2 if 5–25% involved, 3 if 26–50% involved, 4 if 51–75% involved and 5 if > 75% involved. The SQ score is the sum of individual score of each lobe, with a possible range of 0 to 25 (Fig. 2). SQ score of 18 and more was categorized as radiologically severe.

### Statistical analysis

Statistical analysis was performed using the software SPSS (version 24, IBM). Quantitative data are presented as mean, standard deviation, and ranges. Qualitative data are presented as percentages of total. Kappa statistics was determined for each CT scoring system based on categorical variable outcomes (mild vs. severe). Interclass correlation coefficient was used for each scoring system utilizing the continuous variables (absolute value of CT score). *P* value of less than 0.05 was chosen to indicate statistical significance.

## Results

Out of the 115 patients admitted with confirmed COVID pneumonia between June 2020 and December 2020, 57% patients (*n* = 66) were classified as severe. Twenty-six percent (*n* = 30) underwent clinical deterioration resulting in mortality. The median age of the study group was 46 years (IQR 19–73 years) with 63% (*n* = 72) being males. As the initial presenting complaint, 36% (*n* = 41) with cough, 42% (*n* = 48) with fever, and 22% (*n* = 25) with breathlessness.

Sensitivity for the SQ score varied from 85 to 88% (mean 86.5%) while the specificity ranged from 96 to 100%. Sensitivity for the CT-SS test was noted to range from 98.5 to 100% (mean 99.2%) while the specificity ranged from 59 to 65%.

The positive predictive value for SQ test ranged from 96.7 to 100% (mean 98.3%), while the negative predictive value ranged from 83 to 85.5% (mean 85.7%).

The positive predictive value ranged from 77 to 79% (mean 78%), while the negative predictive value ranged from 97 to 100% (mean 98.5%) for the CT SS test.

While adopting a random effects model, there was excellent reliability between the two observers for both SQ and CT-SS, with an agreement of 91.3% and 88%, respectively.

Kappa statistics values for interobserver correlation of 91% and 93% were seen for SQ and CT-SS scores respectively after categorization of the patients into mild and severe COVID cases, again indicating an excellent inter-observer agreement. The extent of this agreement is similar to that found by Jiang et al. [25] for SQ scores (0.91), and by Yang et al. for CT-SS (0.936), thus reiterating the cutoff scores for diagnosing severe COVID pneumonia as 18 for SQ, and 19.5 for CT-SS.

## Discussion

The rapidly evolving global scenario with respect to the current pandemic caused by SARS-CoV-2 has necessitated the orchestrated efforts of various specialties in the diagnosis and treatment of the disease, and radiology holds an essential position in this regard. Though the initial diagnosis is made by way of tests such as the rapid antigen test (RAT) or real-time polymerase chain reaction (rt-PCR), chest radiographs are used for initial severe presentations of pneumonia [10]. CT scan of the chest is reserved primarily for complicated or unresolved pneumonia, or other suspected thoracic pathologies, and is more sensitive to recognizing the pulmonary manifestations of COVID lung disease in comparison to radiographs [26, 27]. In view of this, the proposed CT chest scoring systems, the semiquantitative (SQ) score proposed by Francone et al. [21] and the CT severity score, proposed by Yang et al. [20] have been in worldwide use to help diagnose and prognosticate COVID pneumonia.

In view of the relative nascency of imaging standardisation of COVID lung disease, and the subsequent differing systems of scoring used, it would be prudent to assess the extent of interobserver variability of the above two systems, and to help determine which, if any, more accurately represents the clinical severity and prognosis. To our knowledge, such a study comparing different CT scoring systems for COVID lung disease has not yet been performed.

In our sample population of 115 patients, a majority were males (63%), and percentages of individuals presenting with cough (36%) and fever (42%). Among two experienced radiologists who performed the blinded scoring, sensitivity for diagnosis of severe COVID pneumonia with respect to the SQ score was 85–88%, and 98.5–100% for the CT-SS. This implies a marginally higher sensitivity for CT-SS in identifying severe COVID lung disease. Specificity for recognizing severe COVID lung disease was similar for both scores. The interobserver reliability for both scores was excellent: the interclass correlation when comparing continuous variables (absolute value of score) were 91.3% and 88% for SQ and CT-SS respectively. The interobserver reliability determined via Kappa statistics for categorical variables (mild-moderate vs. severe-critical) were 91% and 93% for SQ and CT-SS, respectively. Limitations of this study include the limited sample size, and subjectivity in assigning scores. This is an issue that has been raised in previous studies as well [28].

## Conclusion

Our study shows that there exists excellent inter-observer agreement for both the SQ and CT-SS scores with regard to the diagnosis of severe COVID lung disease. CT-SS displays marginally higher sensitivity in identifying severe COVID, and may thus be preferred by radiologists as a standard screening tool while assessing CT. The rapid and uniform adoption of this system of scoring may assist in the effective communication of disease severity to referring clinicians as well as between radiologists themselves, for both first-time assessments and for comparison purposes with previous studies to evaluate disease progression. Also, the development of scoring systems based on CT imaging of the chest and the wealth of promising data obtained with regard to correlation of such scores with clinical outcome and laboratory parameters open up new possibilities for further development of novel scoring systems for other infective/inflammatory lung diseases in the future.

## Data Availability

The datasets used and/or analyzed during the current study are available from the corresponding author on reasonable request.

## Abbreviations

COVID-19: Coronavirus disease 2019
SARS-CoV-2: Severe acute respiratory syndrome coronavirus 2
RT-PCR: Real-time polymerase chain reaction
CT: Computed tomogram
SQ: Semiquantitative score
CT-SS: CT severity score
ARDS: Acute respiratory distress syndrome
MOD: Multiple organ dysfunction
MOF: Multi-organ failure
